# Design of Chemoresponsive Soft Matter Using Hydrogen-Bonded Liquid Crystals

**DOI:** 10.3390/ma14051055

**Published:** 2021-02-24

**Authors:** Huaizhe Yu, Kunlun Wang, Tibor Szilvási, Karthik Nayani, Nanqi Bao, Robert J. Twieg, Manos Mavrikakis, Nicholas L. Abbott

**Affiliations:** 1 Robert Frederick Smith School of Chemical and Biomolecular Engineering, Cornell University, 1 Ho Plaza, Ithaca, NY 14853, USA; hy542@cornell.edu (H.Y.); kn428@cornell.edu (K.N.); nb543@cornell.edu (N.B.); 2 Department of Chemistry and Biochemistry, Kent State University, 1175 Risman Drive, Kent, OH 44242, USA; kwang1@kent.edu; 3 Department of Chemical and Biological Engineering, University of Wisconsin−Madison, 1415 Engineering Drive, Madison, WI 53706, USA; tibor.szilvasi@ua.edu

**Keywords:** liquid crystals, chemoresponsive materials, gas sensor, fish freshness

## Abstract

Soft matter that undergoes programmed macroscopic responses to molecular analytes has potential utility in a range of health and safety-related contexts. In this study, we report the design of a nematic liquid crystal (LC) composition that forms through dimerization of carboxylic acids and responds to the presence of vapors of organoamines by undergoing a visually distinct phase transition to an isotropic phase. Specifically, we screened mixtures of two carboxylic acids, 4-butylbenzoic acid and trans-4-pentylcyclohexanecarboxylic acid, and found select compositions that exhibited a nematic phase from 30.6 to 111.7 °C during heating and 110.6 to 3.1 °C during cooling. The metastable nematic phase formed at ambient temperatures was found to be long-lived (>5 days), thus enabling the use of the LC as a chemoresponsive optical material. By comparing experimental infrared (IR) spectra of the LC phase with vibrational frequencies calculated using density functional theory (DFT), we show that it is possible to distinguish between the presence of monomers, homodimers and heterodimers in the mixture, leading us to conclude that a one-to-one heterodimer is the dominant species within this LC composition. Further support for this conclusion is obtained by using differential scanning calorimetry. Exposure of the LC to 12 ppm triethylamine (TEA) triggers a phase transition to an isotropic phase, which we show by IR spectroscopy to be driven by an acid-base reaction, leading to the formation of ammonium carboxylate salts. We characterized the dynamics of the phase transition and found that it proceeds via a characteristic spatiotemporal pathway involving the nucleation, growth, and coalescence of isotropic domains, thus amplifying the atomic-scale acid-base reaction into an information-rich optical output. In contrast to TEA, we determined via both experiment and computation that neither hydrogen bonding donor or acceptor molecules, such as water, dimethyl methylphosphonate, ethylene oxide or formaldehyde, disrupt the heterodimers formed in the LC, hinting that the phase transition (including spatial-temporal characteristics of the pathway) induced in this class of hydrogen bonded LC may offer the basis of a facile and chemically selective way of reporting the presence of volatile amines. This proposal is supported by exploratory experiments in which we show that it is possible to trigger a phase transition in the LC by exposure to volatile amines emitted from rotting fish. Overall, these results provide new principles for the design of chemoresponsive soft matter based on hydrogen bonded LCs that may find use as the basis of low-cost visual indicators of chemical environments.

## 1. Introduction

Responsive soft materials change their structure and properties (e.g., mechanical [1,2], chemical [3] and optical [4]) upon interaction with their environment. This class of soft materials is being widely explored as the basis of a range of emerging technologies, including for drug delivery [5], chemical sensors [3,6], and actuators [1,2]. Liquid crystals (LCs) [7], which are fluid phases within which molecules exhibit long-range order, offer the basis of a range of designs of responsive soft materials, as LCs are able to amplify molecular-level interactions into macroscopic outputs (e.g., changes in shape [8], surface topography [9], rheological properties [10], and optical properties [4,11,12]).

This paper focuses on the design of LCs that respond to their chemical environment. One strategy for the design of chemically-responsive LCs is based on surface-driven anchoring transitions [13]. For example, micrometer-thick films of nematic LCs have been oriented on the surfaces of tailored solids via hydrogen bonding [14], metal cation-ligand coordination interactions [15,16] and bonding to metals (dissociative adsorption) [17]. Disruption of these interactions via competitive binding [16,18], acid-base reactions [19], or redox-reactions [20] by targeted analytes has been shown to lead to LC anchoring transitions. An alternative strategy pursued for the design of chemically responsive LCs involves programming changes in bulk properties of LCs via interactions with analytes. For example, cholesteric LCs formed using reactive chiral dopants have been designed to undergo changes in helical pitch upon reaction with targeted analytes, resulting in changes to Bragg reflections, which can be detected by the naked eye [4,11].

Of particular relevance to the approach reported in this manuscript is a previous report of the use of hydrogen-bonded cholesteric LC polymer films to detect volatile amines [11]. To make the LC polymer network responsive to volatile amines, polymerizable benzoic acid derivatives and a dicarboxylic acid chiral dopant, which induced formation of the cholesteric phase, were incorporated into the polymerized film. When trimethylamine (TMA) penetrated the LC polymer film, an acid-based reaction triggered a change of pitch, thus changing the reflection band and color of the cholesteric film. The authors demonstrated a detection limit of anhydrous trimethylamine (TMA) of 2% (two parts per hundred) after exposure for 120 min (these conditions led to a 7% reduction of the reflection band measured using a UV/Vis spectrophotometer). The dynamic response to TMA accelerated in the presence of water vapor, with a 10% reduction of the reflection band occurring within 10 min when using 2% TMA in water-saturated air. Inspired by this prior work, here we report a study of low molecular weight mesogens containing carboxylic acid groups that form a room temperature nematic phase via dimerization of the carboxylic acids. We show that an acid-based reaction between the LCs and volatile amines triggers a phase transition from a nematic to an isotropic phase that is accompanied by a distinct optical response. This approach yields a LC that responds to volatile amines at the parts per million concentration level within one minute.

Our design of a LC phase formed through hydrogen bonding was guided by past reports [21,22] that benzoic acids dimerize through intermolecular hydrogen bonding, leading to the formation of supramolecular mesogenic species that form LC phases [21,22,23]. In addition, a range of benzoic acid derivatives have been explored, including mixtures with pyridyl or aliphatic groups, and many were shown to form hydrogen bonding LCs through dimerization [24,25,26,27]. However, the nematic temperature ranges of all these hydrogen bonded LCs were much higher than room temperature (90–140 °C) [28]. Although cyclohexyl-based carboxylic acids were synthesized and shown to have lower nematic temperature ranges (50–100 °C) [29], the nematic temperature range still lies well above ambient (during both heating and cooling cycles), limiting their utility for the design of chemoresponsive LC phases that respond at ambient conditions.

The first goal of our study was to determine if it was possible to prepare hydrogen-bonded LCs containing carboxylic acids that exhibited a nematic phase at room temperature. Motivated by the proposal that mixtures of mesogen can lead to a lowering of phase transition temperatures [30], we investigated mixtures of trans-4-pentylcyclohexanecarboxylic acid (C5CA) [29] and 4-butylbenzoic acid (C4BA) [28], which are two known hydrogen bonding LCs. We report characterization of the phase behaviors of mixtures of these two LCs using differential scanning calorimetry (DSC) and polarized optical microscopy (POM). Insights into intermolecular interactions (e.g., homodimer versus heterodimer formation) that underlie the phase behavior are obtained using Fourier Transform Infrared Spectroscopy (FTIR) and first-principles calculations.

The second goal of our study was to determine if it was possible to take advantage of the hydrogen bonded LCs formed using carboxylic acids to report the presence of organoamines that undergo acid-base reactions. We used triethylamine (TEA) in our experiments as a model base because TEA is commonly used as a simulant of key volatile amines emitted by spoiled fish or meat [31,32]. We report TEA to trigger nematic-to-isotropic phase transitions at room temperature and use both experimental methods and electronic structure calculations to test the hypothesis that TEA triggers the phase transition by disrupting hydrogen bonding. We also characterize the complex kinetic pathway that we observed to underlie the phase transition with the longer term goal of using descriptors of the spatiotemporal patterns to develop machine-learning approaches to analyze the response of the LC [33,34].

The third goal of our study was to explore the chemical specificity of the above-described phase transition induced by TEA, with a particular focus on water due to its ubiquitous presence in contexts relevant to food safety. We sought to determine if hydrogen bonding interactions between water (or other hydrogen bonding species) and the carboxylic acid groups of the LC would impact the phase behavior of the LC. As a proof of concept experiment, we also explored the response of the LC to complex mixtures of volatile amines, such as cadaverine and putrescine (diamines), emitted by rotting fish [35]. We sought to determine if these organoamines triggered phase transitions of the hydrogen bonded LC reported in our study.

## 2. Materials and Methods

### 2.1. Materials

The 4-*n*-butylbenzoic acid (C4BA) and trans-4-*n*-pentylcyclohexanecarboxylic acid (C5CA) were purchased from Combi-Blocks (San Diego, CA, USA). Triethylamine was bought from Sigma-Aldrich (Milwaukee, WI, USA). Nitrogen gas (99.998% purity) was purchased from Airgas (Radnor Township, PA, USA). Fischer’s Finest glass slides were purchased from Fischer Scientific (Pittsburgh, PA, USA). We used SU-8 2050 and developer, both of which were obtained from MicroChem (Westborough, MA, USA). Spacers (glass fibers with diameters of 5 μm) were obtained from EM industries, Inc (Hawthorne, NY, USA). All solvents were commercial-grade and used as purchased. All aqueous solutions used in this study were prepared using deionized water possessing a resistivity greater than 18.2 MΩ.

### 2.2. Representative Procedure Used to Synthesize the H-Bonded Liquid Crystal Mixture

To a 5 mL sample vial fitted with a magnetic stirrer, we added the two carboxylic acid components in a desired molar ratio, and then added 2 mL of ethyl acetate to dissolve the mixture. The mixture was kept under vacuum at room temperature for 12 h to remove the solvent.

### 2.3. Preparation of LC Thin Films on Glass Substrates

Polymeric microwells (200 μm in diameter and 5 μm in depth) were fabricated using previously reported procedures [36]. Approximately 2 μL of LC was micropipetted into each microwell. A microcapillary was touched to the surface of the microwell to remove any excess LC.

### 2.4. Fourier Transform Infrared Spectroscopy (FTIR)

IR spectroscopy was performed in transmission mode using a Bruker Vector 33 FTIR spectrometer (Bruker Optics Inc, Billerica, MA, USA). Spectra were acquired at 4 cm^−1^ resolution using 32 scans. OPUS software was used for analysis. The path length of each sample was 1 mm.

### 2.5. Characterization of LC Phase Behavior

LC phase behavior was characterized in optical cells prepared from two glass slides. Glass spacers with diameters of 5 μm were used to define the distance between the two surfaces of the glass slides. The C4BA-C5CA mixture was drawn into the optical cell as an isotropic phase (115 °C < T < 120 °C). A polarized light microscope (Olympus BX-60) was used to observe the LC phase behavior.

### 2.6. Differential Scanning Calorimetry (DSC)

LC phase behavior was also determined using DSC (2920 Modulated DSC, TA Instruments Inc., New Castle, DE, USA). The heating/cooling rate was 5 °C/min. Thermal Advantage software from TA Instruments was used for analysis.

### 2.7. Measurement of Optical Retardance

The optical retardance (Δ*r*) values of LC films were measured using a Berek compensator (Olympus, Melville, NY, USA). Values were measured at five locations in each sample and were averaged. We calculated the effective birefringence (Δ*n*_eff_) as Δ*n*_eff_ = Δ*r*/Δ*d*, where the film thickness is Δ*d*. The tilt angle (*θ*, measured from the surface normal) was calculated using [37]

Δ*r* ≈ *n*_o_*n*_e_/(*n*_o_^2^sin^2^(*θ*) + *n*_e_^2^cos^2^(*θ*))^0.5^ − *n*_o,_ (1)

where *n*_e_ and *n*_o_ are the extraordinary and ordinary refractive indices of the LC, respectively.

### 2.8. Phase Transitions Triggered by TEA

A stream of nitrogen containing TEA was flowed across LC hosted in microwells using the set-up shown in Figure S1 [38]. Nitrogen gas containing 120 ppm TEA, a concentration that was confirmed by gas chromatography (Figure S2), was generated using a bubbler (bubbling of N_2_ through liquid TEA), and diluted to 12 ppm using nitrogen. The gas flow rate was set at 1000 mL/min by using rotameters (Aalborg Instruments & Controls, Inc., Orangeburg, NY, USA).

### 2.9. Computational Methods

All calculations were carried out using Gaussian 09 version D.01 [39]. We employed the Perdew-Burke-Ernzerhof (PBE) density functional [40] and def2-SVP basis set [41] for geometry optimization while final energies were obtained using the M06-2X functional [42] with def2-TZVP basis set [41] with the previously optimized geometries. Previously, we applied this strategy to reduce computational cost but maintain high accuracy and successfully described LC-based systems in agreement with experiments [16,20,43,44,45,46]. We used Grimme’s D3 empirical dispersion correction in all calculations to correct the known limitation of density functionals to describe dispersion interactions [47]. Additionally, Truhlar’s Solvation Model D (SMD) was employed to account for the effect of the liquid phase using parameters developed for acetic acid [48]. Therefore, the overall computational method can be abbreviated as M06-2X-D3(SMD = acetic acid)/def2-TZVP//PBE-D3(SMD = acetic acid)/def2-SVP. Additionally, counterpoise correction was employed in all reaction energy calculations. IR calculations were performed using the optimized geometries at the same level of theory. Calculated IR frequencies are often shifted from experimental values and thus empirical correction factors are used to obtain closer agreement between experiments and calculations [20]. We applied an empirical correction factor of 1.013 to calculated IR frequencies.

Reaction energies (ΔE) are formally calculated as ΔE = ΣE_Products_ − ΣE_Reactants_, where ΣE_Reactants_ is the sum of the total energy of the reactants and ΣE_Products_ is the sum of the total energy of the products. Negative values of ΔE indicate that the reaction is energetically favored. To reduce the conformational search problem for the aliphatic tail of C4BA and C5CA, we used the surrogate molecules 4-methylbenzoic acid (C1BA) and 4-methylcyclohexanecarboxylic acid (C1CA), respectively, in all calculations. Our test calculations suggest that this simplification does not have a significant effect on the calculated properties (IR and ΔE).

## 3. Results and Discussion

### 3.1. Characterization of Hydrogen-Bonded LCs with Carboxylic Acid Groups

**Scheme 1:**
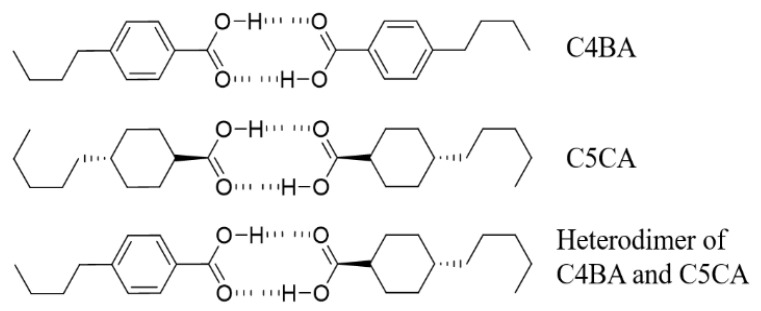
Molecular structures of the 4-butylbenzoic acid (C4BA) dimer, trans-4-pentylcyclohexanecarboxylic acid (C5CA) dimer, and heterodimer of C4BA and C5CA.

**Figure 1:**
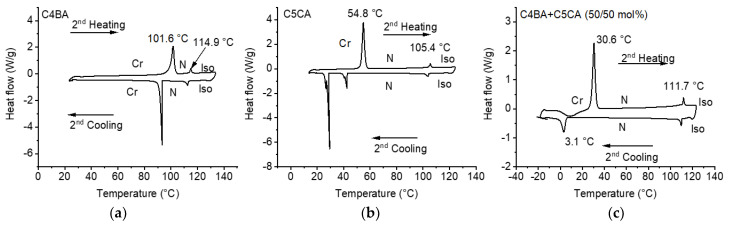
Differential scanning calorimetry (DSC) plots of (**a**) C4BA, (**b**) C5CA, and (**c**) heterodimers of C4BA and C5CA (mole ratio of 1:1). The upper line corresponds to the heating line and the bottom line to cooling, as indicated by the arrows. DSC scan rate is 5 °C/min. Cr, N and Iso indicate crystal, nematic, and isotropic, respectively.

**Figure 2:**
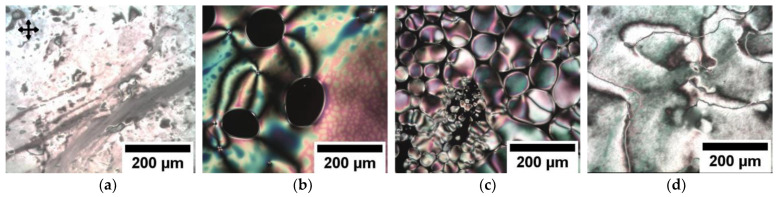
Optical micrographs (crossed-polars) of the phase textures of the LC mixture containing 50/50 mol% of C5CA/C4BA at (**a**) 32.0 °C when heating, (**b**) 108.0 °C during heating, (**c**) 108.3 °C during cooling, and (**d**) 37.0 °C during cooling. The orientations of polarizers used to obtain all images in Figure 2 are indicated by the black arrows in (**a**).

The phase transition temperatures of the two pure compounds C4BA and C5CA (Scheme 1) were measured using DSC and POM. A summary of transition temperatures and enthalpies is shown in Table S1. Inspection of Figure 1 shows that the crystal-to-nematic (T_Cr-N_) and nematic-to-isotropic (T_N-Iso_) transition temperatures of C4BA and C5CA were 101.6 °C, 114.9 °C, and 54.8 °C, 105.4 °C, respectively. These transition temperatures are consistent with past studies [28,29,49]. The lower T_Cr-N_ of C5CA (as compared to C4BA) is due to the cyclohexyl ring in C5CA, as reported in previous work [50]. However, neither of these two compounds exhibits a nematic phase at room temperature. Motivated by past reports of melting point depression of mixtures [30], we mixed C5CA and C4BA with a 1:1 molar ratio. We observed that the LC mixture melted at 30.6 °C and became isotropic at 111.7 °C during heating (Figure 1c), and then reformed a nematic phase at 110.6 °C and recrystallized at 3.1 °C during cooling. Similar conclusions were reached by using POM (Figure 2). Additionally, after cooling, the mixture confined within microwells remained as a nematic phase at room temperature (25 °C) for more than five days. The long-lived nematic state enabled the experiments reported in this paper aimed at exploring the chemoresponsive properties of the LC at ambient conditions.

As shown in Figure 1c, the presence of a single peak at 30.6 °C in the thermogram is consistent with the LC mixture forming a heterodimer, since homodimers of C4BA or C5CA possess distinct T_Cr-N_ at 101 °C or 54.8 °C, respectively, as shown in Figure 1a,b. To test this interpretation, we performed DSC using LC mixtures containing 25/75 mol% C4BA and C5CA. As shown in Figure S3, when C5CA is present in excess in the mixture, T_Cr-N_ of this LC mixture is at 29.6 °C, which is consistent with the T_Cr-N_ of C4BA/C5CA (50/50 mol%) mixture, indicating the existence of heterodimers in the C4BA/C5CA (25/75 mol%) mixture. In contrast to the C4BA/C5CA (50/50 mol%) mixture, the DSC trace of the C4BA/C5CA (25/75 mol%) mixture showed a second phase transition at 42.1 °C. This second phase transition occurred at a temperature that was lower than the T_Cr-N_ of C5CA homodimers (54.8 °C). However, past studies have revealed that pure component melting points are often downshifted in temperature when measured in mixtures [30,50], and thus we interpret the peak at 42.1 °C to likely correspond to the melting of C5CA homodimers in the mixture.

**Figure 3:**
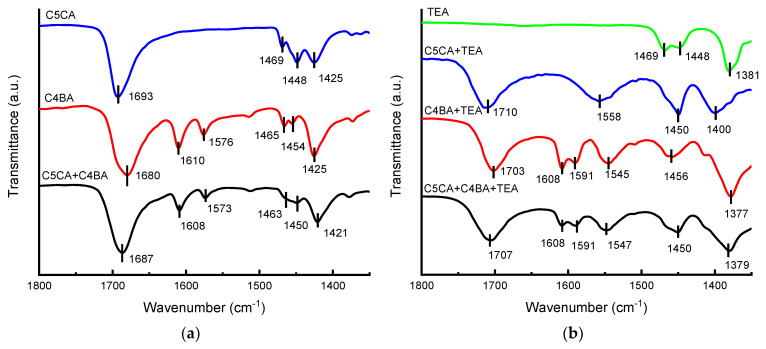
FTIR spectra of (**a**) C5CA, C4BA, and C4BA/C5CA (50/50 mol%) and (**b**) TEA, and mixtures of C5CA/TEA, C4BA/TEA and C5CA/C4BA/TEA.

To provide insight into the molecular interactions underlying the formation of heterodimers of C4BA/C5CA (50/50 mol%), we performed IR spectroscopy (Figure 3a) to characterize the C=O stretching vibration (see Table 1 for a summary of the peak assignments). Past studies have established that the C=O vibrations are distinct for dimeric and monomeric carboxylic acid groups [22]. As shown in Figure 3a, we first measured the IR spectra of pure C5CA and C4BA, revealing C=O peaks at 1693 and 1680 cm^−1^, respectively. This observation is consistent with prior reports of 1678 to 1694 cm^−1^ for dimeric species [51]. In contrast, the C=O stretching vibration of a monomeric carboxylic acid in dilute solutions is found at 1760 cm^−1^ [23].

The formation of carboxylic acid homodimers was further validated by first-principles calculations, which confirmed that the C=O stretching peaks of dimerized C1CA (surrogate of C5CA) and C1BA (surrogate of C4BA) lie at 1693 and 1680 cm^−1^, while C=O stretching peaks of monomeric C1CA and C1BA lie at 1747 and 1765 cm^−1^, respectively. Additionally, we note that both experiments and simulations show that the stretching frequency of the C=O group of C5CA is higher than that for C4BA by 13 cm^−1^.

To evaluate the thermodynamic driving force for the formation of homodimers from monomers, we carried out DFT calculations, which revealed that dimerization of C1BA or C1CA (C4BA or C5CA surrogates) is energetically favored by −47 or −45 kJ/mol, respectively. We note that a difference of 6 kJ/mol in reaction energy corresponds to a one order of magnitude difference in equilibrium constant at room temperature. Therefore, the calculated ~−46 kJ/mol reaction energy suggests that all monomers form homodimers in the pure LC.

Next, we performed IR measurements of the LC mixture containing 50/50 mol% of C5CA/C4BA. Inspection of Figure 3a reveals that the C=O group has a transition dipole moment at 1687 cm^−1^, which lies between the peak positions at 1680 and 1693 cm^−1^ for homodimers of C4BA and C5CA, respectively, indicating formation of heterodimers between C5CA and C4BA (Scheme 1). Additionally, the full width at half maximum (FWHM) of the C=O vibration for LC mixtures is 38 cm^−1^, which is between the FWHM of C5CA homodimer (34 cm^−1^) and C4BA homodimer (44 cm^−1^), thus confirming the formation of heterodimer. To validate this interpretation, calculations were carried out for the C=O group in the C1CA/C1BA heterodimer. In agreement with experimental peak position of 1687 cm^−1^ measured using the C5CA/C4BA mixture, simulation shows that the peak corresponding to C=O group of C1CA/C1BA heterodimer lies at 1689 cm^−1^. Thus, both experimental and computational IR results support formation of heterodimers of carboxylic acids in C5CA/C4BA mixture.

We also calculated the formation energy of the heterodimer (C1BA and C1CA) from monomers to be −46 kJ/mol. For comparison, as we discussed above, the formation energy of C1BA (C1CA) homodimers is −47 (−45) kJ/mol. Thus, the formation of heterodimers from homodimers is energetically neutral. However, our calculation does not capture collective effects such as the entropy of mixing, which would favor the formation of heterodimers over homodimers. Therefore, our results lead to the proposal that heterodimer formation is an entropy-driven process. Overall, the combined experimental and computational results above provide support for the conclusion that C5CA and C4BA largely form heterodimers in 1:1 mixtures, as shown in Scheme 1.

### 3.2. Experimental Characterization of Influence of TEA on the Hydrogen-Bonded LCs

**Figure 4:**
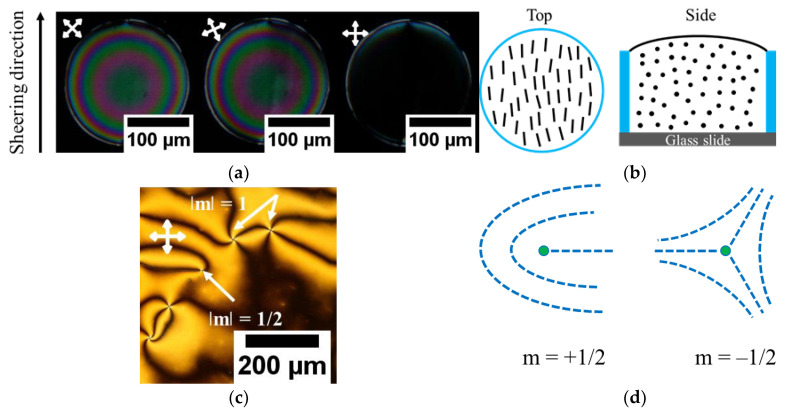
(**a**) Optical micrographs of a C5CA/C4BA mixture confined to a 5 μm-deep microwell. (**b**) Top view and side view of LC orientation as interpreted from the images in (**a**). (**c**) LC mixture sandwiched between two glass films. (**d**) shows a schematic illustration of the LC director near +1/2 and −1/2 defects. Orientations of polarizers are indicated by the white arrows.

Acid-base reactions of amines and carboxylic acids have been widely characterized in isotropic phases [55]. We explored the influence of TEA on the LC phase behavior exhibited by a 1:1 mixture of C5CA and C4BA guided by the hypothesis that TEA would undergo an acid-base reaction with the carboxylic acid dimers that formed the LC. Past studies [56] have also reported that the thermodynamic driving forces for acid-base reactions are sufficiently large that they can disrupt hydrogen bonding interactions. Accordingly, we speculated that TEA would react with the carboxylic acids of the C5CA/C4BA mixture, thus breaking the hydrogen bonding that leads to the formation of the mesogenic species and causing a transition to an isotropic phase. To test this proposal, we first deposited nematic C5CA/C4BA mixtures into microfabricated wells (depth of 5 μm) with a borosilicate substrate. We observed that a largely uniform director profile across the entire film thickness could be induced by shearing the top surfaces of the LC within the microwells with a pipette tip (vertical in Figure 4a). The uniformity of the director profile induced by this treatment was determined by analyzing optical images of the LC while rotating the sample between crossed polars. Specifically, as shown in Figure 4a, we observed extinction of the transmitted light when either the polarizer or analyzer was orientated parallel to the shearing direction (vertical for Figure 4a) of the LC mixture; at other relative orientations of the sample and polars, we observed transmission of light. At the edge of the microwells, we observed the LC to exhibit a bright appearance even when the polarizer was oriented along the shearing direction, indicating that the LC was strained locally by interaction with the side-walls of the microwells.

Next, we characterized the LC within an optical cell formed by two borosilicate surfaces (Figure 4c). Inspection of Figure 4c reveals the presence of dark brushes, which correspond to regions of the LC where the relative orientations of the LC director and crossed polars lead to the extinction of transmitted light. Accordingly, in these regions, the director lies either parallel or perpendicular to the polarizer or analyzer. The points where two brushes meet correspond to line disclinations of strength m = ±1/2 (Figure 4c). The presence of defects with strength m = ±1/2 indicates that the anchoring of the LC mixture at the borosilicate surface is planar. We measured the retardance of the LC film to be 503 ± 53 nm. Accordingly, the birefringence (Δn) of the LC mixture was calculated to be 0.10 ± 0.01 from the ratio of the measured retardance and thickness of the LC film (5 μm). For comparison, 4-(trans-4-pentylcyclohexyl) benzonitrile, which also contains one phenyl and one cyclohexyl ring, exhibits a birefringence of 0.12 at room temperature [57].

**Figure 5:**
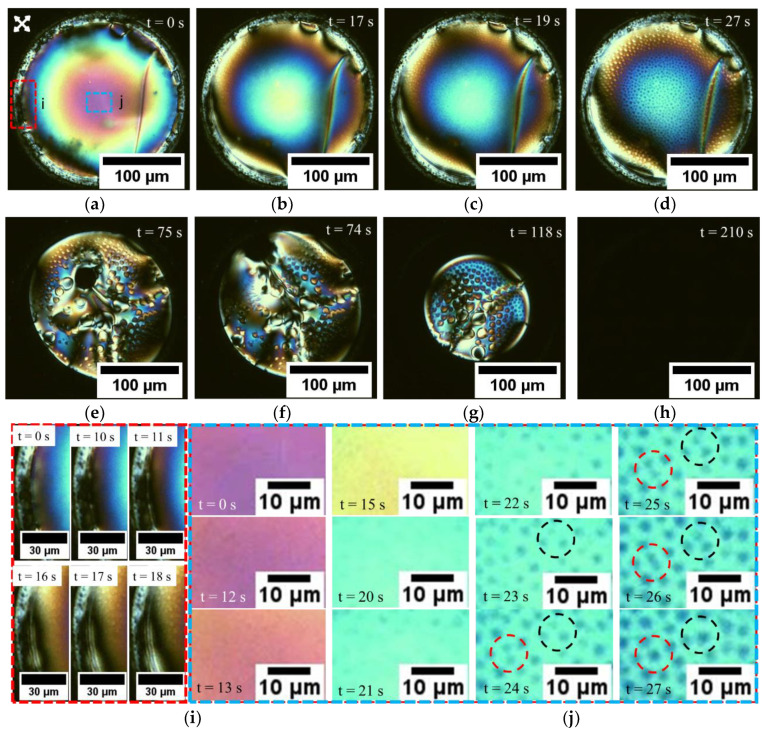
Representative optical micrographs of a C5CA/C4BA mixture hosted within a microwell exposed to a vapor of 12 ppm TEA as a function of time (**a**–**h**). (**i**,**j**) show enlarged images of the circled area in (**a**). The TEA stream flows from left to right. The LC within the microwell was sheared in the vertical direction, and the crossed polars are oriented along the diagonals of the images in (**a**–**h**).

To characterize the anchoring of the hydrogen bonded LC at the LC-air interface, the LC mixture was deposited into a TEM grid supported on a borosilicate surface. The top surface of the LC was exposed to air with 30% relative humidity (RH). We measured the optical retardance of the LC film (Δn × d, d = 20 μm) to be 1744 ± 97 nm, which corresponds to a birefringence of 0.09 ± 0.01, and is thus consistent with planar anchoring at the LC-air interface. We also investigated whether the humidity of the air influenced the optical retardance of the LC film. We found there to be no measurable change in optical retardance when the LC film was exposed to 30% RH and 0% RH. We note the appearance of a line disclination in some LC samples after shearing (e.g., Figure 5a), which hints that the LC may relax to assume a tilted orientation over time. Additional studies are needed to understand this time-dependent behavior.

To explore the response of the hydrogen bonded LC mixture to TEA, we deposited the mixture into the microwells as an isotropic phase at 120 °C, and then cooled the samples to room temperature (25 °C). The LC mixture was subsequently exposed to a N_2_ stream containing 12 ppm TEA at a flow rate of 1000 mL/min, and the optical appearance was recorded between crossed polarizers (Figure 5). The direction of flow of the gas is from left to right in Figure 5. Inspection of Figure 5 reveals that exposure of the LC sample to TEA resulted in a progression of optical states: first, we observed a change in color of the LC film; second, we observed the formation and coalescence of domains; finally, the LC mixture assumed a dark state. Below we discuss each of these states of the LC film.

The above-described changes in optical appearance of the LC film that are triggered by exposure to TEA are proposed to be caused by an acid-base reaction between the carboxylic acid groups of C5CA and C4BA and TEA, thus disrupting the dimers that underlie the formation of the LC phase and leading to a transition to an isotropic phase. To characterize the final dark state (Figure 5h) of the mixture following exposure to TEA, we used conoscopic polarized light microscopy. In contrast to a homeotropically orientated LC, which generates an interference pattern consisting of a dark cross overlying concentric rings (Figure S4a), conoscopic examination of the sample shown in Figure 5h generated a dark featureless image (Figure S4b), thus confirming formation of an isotropic phase. To provide additional insight into the origins of the transition from the LC phase to the isotropic phase, we performed FTIR of a mixture of heterodimers that were exposure to TEA. As shown in Figure 3b, the TEA-triggered shift in the position of the peak corresponding to C=O stretching (from 1687 cm^−1^ to 1707 cm^−1^), when combined with the appearance of a peak corresponding to asymmetric stretching of COO^−^ at 1547 cm^−1^, indicate the formation of carboxylates. Additionally, we mixed either C5CA or C4BA with TEA and measured the FTIR spectrum. As shown in Figure 3b, a blue shift of the C=O stretching of around 20 cm^−1^ and the appearance of peak at 1558 and 1545 cm^−1^ confirmed the formation of carboxylates via acid-base reaction.

We also performed DFT calculations to understand the reaction between the carboxylic acid dimers and TEA. We found that the reaction between the C1BA (C1CA) dimer and TEA, to form carboxylates and protonated amine, is highly exothermic by −83 (−73) kJ/mol, which we would expect to lead to a fast and complete acid-base reaction resulting in ion pairs. Additionally, we also calculated the IR signatures of the C1BA^−^[H-TEA]^+^ and C1CA^−^[H-TEA]^+^ ion pairs, which revealed a new IR peak involving the COO^-^ moiety at 1541 and 1562 cm^−1^, respectively, in close agreement with the experimentally observed peaks (Figure 3b and Table 1). Thus, our experimental characterization and computational results together support our conclusion that the overall response to TEA is a phase transition of the LC mixture to an isotropic phase that is caused by an acid-base reaction between C5CA, C4BA, and TEA.

As noted above, while the overall response of the LC to TEA is a phase transition, the initial evidence of the LC optical response was a change in color of the LC film (Figure 5). We interpret the change in color of the film (under white light illumination) to arise from a change in optical retardance. By comparing Figure 5i,j, we conclude that the change in optical retardance starts first at the leading edge (11 s after exposure) of the LC film within the microwell. Specifically, Figure 5i shows that the initial change in optical retardance occurs after 11 s of exposure to 12 ppm TEA, with a corresponding change in color from blue (corresponding to a retardance of 600 ± 50 nm) to orange (retardance of 480 ± 50 nm). In contrast, Figure 5j reveals that a change of optical retardance in the center of the LC film was evident after 13 s of exposure to TEA, with a change in color from magenta (1100 ± 50 nm; the larger initial retardance value recorded at the center of the sample as compared to the edge reflects the convex shape of the meniscus of the film) to orange (950 ± 50 nm) to yellow (800 ± 50 nm) to cyan (680 ± 50 nm) from 13 to 21 s. As discussed above, we expect that the TEA reacts rapidly with the carboxylic acid dimers to form an isotropic ammonium carboxylate phase. For this reason, we speculate that the decrease of optical retardance described above is due to the formation of an isotropic film rich in ammonium carboxylate that overlies the LC film. We cannot, however, also rule out that some TEA diffuses into the LC film, and locally lowers the orientational order of the nematic phase.

Inspection of Figure 5i,j also reveals, following the initial and spatially uniform change in interference color, the formation of small and randomly dispersed domains. These domains were evident first at the leading edge of the LC film (18 s) and then at the center (21 s) of the microwells. The domains located at the center of the sample were observed to have a lower retardance (blue, 600 ± 50 nm) than the surrounding film (cyan, 680 ± 50 nm). Since the product of the reaction between the LC mixture and TEA is an isotropic phase, we interpret these domains to comprise isotropic phases. Additional observations (see domains within dashed circles in Figure 5j) revealed that the domains were mobile, and that they coalesced with each other when they were larger than about 2 μm in diameter. The process of coalescence was observed to continue until the domains were sufficiently large that they spanned the film and thus caused the film to assume a dark appearance. We also observed the isotropic domains to migrate towards the edge of the sample, thus creating a net flux of the isotropic phase towards the edge of the microwells (Figure 5e,f). As isotropic domains accumulated at the edges of the microwells, the nematic regions in the center of the microwells decreased in size (Figure 5g). The final state of the mixture was a uniform isotropic phase, as described above. Overall, these observations reveal that the LC mixture is able to amplify an acid-base reaction from the atomic-scale into the optical changes via a complex hierarchy of spatial and temporal processes.

### 3.3. Selectivity and Reversibility

Next, we sought to determine if the phase transition described above (and shown in Figure 5) was selective to TEA. Specifically, we aimed to determine if hydrogen-bonded species such as water can compete with dimer formation to trigger phase transitions within the nematic phase. As shown in Figure S5a, exposure of the LC mixture to a stream of air with 80% RH at 1000 mL/min did not trigger a phase transition after one hour of exposure. As shown in Figure S5b, the DSC trace obtained after humid air exposure is the same as that shown in Figure 1c. This result is consistent with the low solubility of aromatic carboxylic acids in water, such as C4BA and C5CA. For example, benzoic acid has a solubility in water of only 3 g/100 g [58]. Additionally, past studies show that dimerization of carboxylic acids is energetically favored in aqueous solution [59,60,61].

We also performed DFT calculations to explore the effect of water on carboxylic acid dimerization. We found that the interaction energy between C1BA (C1CA) and a water molecule is only −24 (−22) kJ/mol, while the water dimer formation energy is −12 kJ/mol, and the C1BA (C1CA) dimer formation energy is −47 (−45) kJ/mol. Thus, we conclude that the carboxylic acid dimer formation is still energetically favorable even if water is present in the system. Overall, both experiments and computations demonstrate that water cannot break the hydrogen bonding in the LC.

We investigated the selectivity of our LC-based system to TEA relative to other chemical species such as gaseous N_2_, dimethyl methylphosphonate (DMMP), ethylene oxide, and formaldehyde (the concentrations of the latter three gases were 10 ppm). None of the selected gases triggered a phase transition of the LC mixture. This result is consistent with a selectivity based on an acid-base reaction.

To test if the phase transition triggered by TEA was reversible, we exposed a TEA-treated (isotropic) sample of C5CA/C4BA to a gaseous nitrogen stream. We observed the film to undergo a phase transition from the isotropic phase back to a nematic phase after 5 min. To confirm that the phase transition from the isotropic to nematic phase was due to diffusion of TEA out of the mixture, we measured the FTIR spectrum of the C5CA/C4BA mixture after N_2_ exposure. The IR spectrum was the same as that shown in Figure 3a (C5CA + C4BA). The C=O stretching peak blue-shifted back to 1687 cm^−1^ and the COO^-^ peak at 1547 cm^−1^ disappeared, indicating that the carboxylic acids reformed dimers via hydrogen bonding.

### 3.4. Response to Complex Mixtures of Amines from Rotting Fish

**Figure 6:**
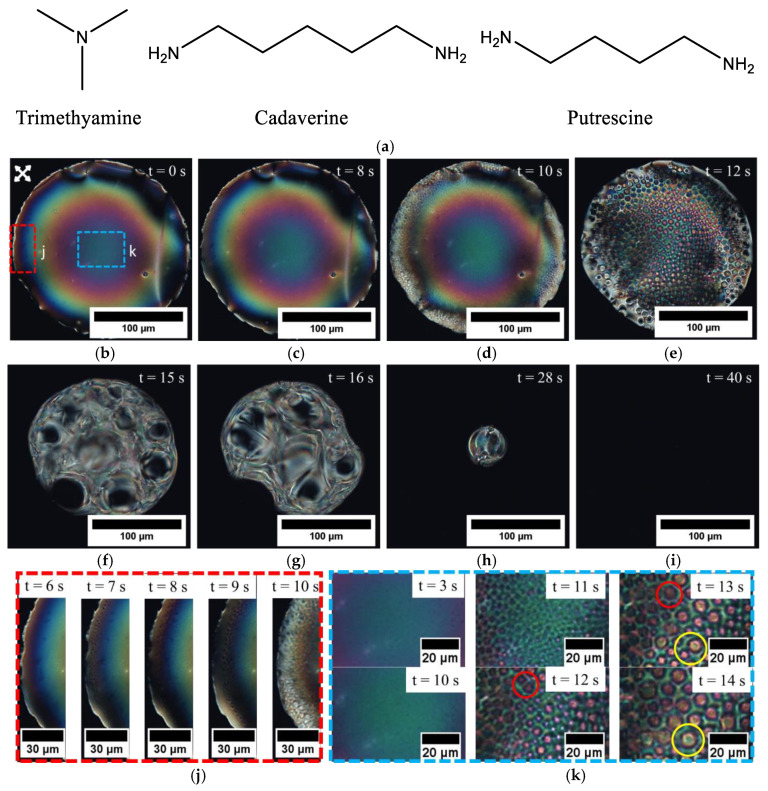
(**a**) Molecular structures of trimethylamine, cadaverine and putrescine. Representative optical micrographs of a C5CA/C4BA mixture hosted within a microwell exposed to volatile amines emitted from Tilapia fish at different times (**b**–**i**). (**j**,**k**) show enlarged images of the area circled in (**b**). The crossed polars are oriented along the diagonals of the images in (**b**–**i**) and the LC was sheared in the vertical direction.

TEA is used as a simulant of key volatile amines (Figure 6a), such as trimethylamine (TMA) and diamines (cadaverine and putrescine), emitted by spoiled fish or meat [31,32,35]. To determine the relevance of the findings reported in this paper to the design of responsive LCs with potential utility for monitoring the freshness of fish, we placed 2 g of fresh Tilapia fish and the LC mixture in the same chamber and recorded the optical appearance of the LC mixture. We observed no change in the optical appearance of the LC over 30 min (Figure S6a). Next, we stored 2 g of Tilapia fish at ambient temperatures for 2 days and then exposed a LC sample to the fish. As shown in Figure 6, we observed the LC to undergo an almost immediate response to volatiles emitted from the fish. In contrast to the LC response to TEA, however, the initial response evident in the LC was not a color change but the formation of domains of isotropic mixture. The isotropic domains appeared at the edge of the sample within 7 s of exposure to the fish. Similar to the TEA response, the domains coalesced with each other to form larger domains, which subsequently migrated to the edge of microwells. The final dark state of the LC sample (Figure 6i) was confirmed by conoscopic polarized microscopy to be an isotropic sample. As an additional control experiment, we placed a piece of 2 cm by 2 cm paper towel saturated with water in the exposure chamber along with a LC film. As shown in Figure S6b, the water that evaporated from the towel did not trigger any optical changes in the LC within 30 min. Overall, these initial results suggest that the LC mixture reported in this paper responds quickly to volatile species generated by rotting fish.

We end by noting that both a past study [11] and our study use liquid crystalline materials containing carboxylic acid groups to detect volatile amines through acid-base reactions. We note two key differences between the two studies. First, the prior study [11] uses a polymeric network doped with chiral dicarboxylic acids, whereas our study employed LCs based on hydrogen bonded dimers (no polymer network). Second, the prior study reported an optical response based on a change of pitch of the cholesteric LC, whereas the response of the system we have studied is dominated by a phase transition from a nematic to an isotropic phase. While the potential merits of using a LC polymer network is high in terms of materials integration (e.g., creation of a colorimetric film that responds to volatile amines), our results suggest that the polymer network may also inhibit the responsiveness of the material. Although there are several key differences between the designs of the two experimental systems, we note that the LCs based on hydrogen bonded dimers respond to 12 ppm of volatile amine within a minute, whereas the polymerized LC film responded to parts per hundred concentrations in approximately 10 min. We comment, however, that additional studies are needed to understand how polymer networks impact the design of chemoresponsive LCs, as recent studies suggest that their influence on responsiveness is complex and not easily anticipated [62].

## 4. Conclusions

In summary, we have designed a LC system that provides a visible and reversible optical response to volatile organoamines. The design is based on hydrogen bonded heterodimers of two carboxylic acids that we have found to exhibit a long-lived nematic LC phase at room temperature. Both experiment (optical microscopy, DSC and FTIR) and computations reported in this paper demonstrate that an acid-base reaction between TEA and the carboxylic acids disrupts hydrogen bonding within the mixture, which is responsible for the formation of the LC phase, and leads to a phase transition. We show that the LC responds to 12 ppm of TEA within 10 s and to volatiles emitted from two-day old fish within a few seconds. Overall, these results provide new principles for the design of chemoresponsive soft matter based on hydrogen bonded LCs that may find use as the basis of low-cost visual indicators of chemical environments (e.g., within packaged food).

Our results also generate a range of future directions of inquiry. For example, our results show that the volatile amines generate information-rich optical outputs from hydrogen bonded LCs, responses that involve the nucleation, growth, coalescence and migration of isotropic domains. We predict that machine learning approaches can likely be developed to interpret the spatial and temporal characteristics of these complex responses [33] to provide additional information regarding the identity of chemical species present and their concentrations. This direction of research is ongoing and will be reported elsewhere.

## Figures and Tables

**Table 1:** Vibrational frequency (cm^−1^) assignments for peaks in Figure 3. [52,53,54] Computational results are listed in parentheses.

Frequency	C4BA	C5CA	C4BA + C5CA	TEA
ν(C=O)	1680 (1680)	1693 (1693)	1687 (1689)	
ν(C-C)_ring_	1610, 1576		1608, 1573	
β(O-H)	1425	1425	1421	
ν_as_(COO^−^)	1545 (1541)	1558 (1562)	1547	
β(C-H)	1465, 1454	1469, 1448	1463, 1450	1469, 1448
ν(C-N)				1381

ν: indicates stretching, β: indicates bending, “as”: indicates asymmetric.

## Data Availability

The data presented in this study are available on request from the corresponding author.

## Supplementary Materials

The following are available online at https://www.mdpi.com/1996-1944/14/5/1055/s1. Figure S1: Schematic illustration of the flow cell used to expose a supported LC film to a gas stream at specified flow rate, concentration of TEA, Figure S2: Relationship between TEA concentration and gas chromatography signal. The red dot indicates the concentration flowing in the F1 stream, Figure S3: Differential scanning calorimetry (DSC) plots of a mixture of 25 mol% C4BA and 75 mol% C5CA. The upper line corresponds to heating and the bottom line to cooling. DSC scan rate was 5 °C/min, Figure S4: Conoscopic polarized light micrographs (crossed polarizers) of (a) a LC film with a uniform homeotropic orientation, (b) a film of the isotropic 1:1 mixture after TEA (12 ppm) exposure, Figure S5: (a) Optical micrographs of representative microwells containing the C4BA+C5CA mixture after exposure to 80% RH air (same as initial state). (b) Differential scanning calorimetry (DSC) plots of 50 mol% C4BA and 50 mol% C5CA mixture after exposure to humid air. The upper line corresponds to heating and the bottom line to cooling. Scale bar: 100 μm. DSC scan rate 5 °C/min, Figure S6: Optical micrographs (crossed polars) of representative microwells containing the C4BA+C5CA mixture (a) after contacting fresh fish (b) after contacting a paper towel saturated with water. The final states of both samples were indistinguishable from their initial states, Table S1: Transition temperatures (T, °C) and enthalpies (ΔH, J/g) of LCs obtained by DSC in the heating cycle.
