# Supplementary material for: Design of Chemoresponsive Soft Matter Using Hydrogen-Bonded Liquid Crystals
Source: Materials (Basel). 2021 Feb 24;14(5):1055. doi: 10.3390/ma14051055 (PMC7975976; doi:10.3390/ma14051055)
Supplement: Supplementary file 1 [file materials-14-01055-s001.pdf]

Supplementary Materials: Design of Chemoresponsive Soft Matter using Hydrogen-Bonded Liquid Crystals

Huaizhe Yu [1], Kunlun Wang [2], Tibor Szilvási [3], Karthik Nayani [1], Nanqi Bao [1], Robert J. Twieg [2,*], Manos Mavrikakis [3,*], and Nicholas L. Abbott [1,*]

1 Robert Frederick Smith School of Chemical and Biomolecular Engineering, Cornell University, 1 Ho Plaza, Ithaca, NY 14853, USA; hy542@cornell.edu (H.Y.); kn428@cornell.edu (K.N.); nb543@cornell.edu (N.B.)

2 Department of Chemistry and Biochemistry, Kent State University, 1175 Risman Drive, Kent, OH 44242, USA; kwang1@kent.edu

3 Department of Chemical and Biological Engineering, University of Wisconsin–Madison, 1415 Engineering Drive, Madison, WI 53706, USA; tibor.szilvasi@ua.edu

* Correspondence: rtwieg@kent.edu (R.J.T.); emavrikakis@wisc.edu (M.M.); nabbott@cornell.edu (N.L.A.)

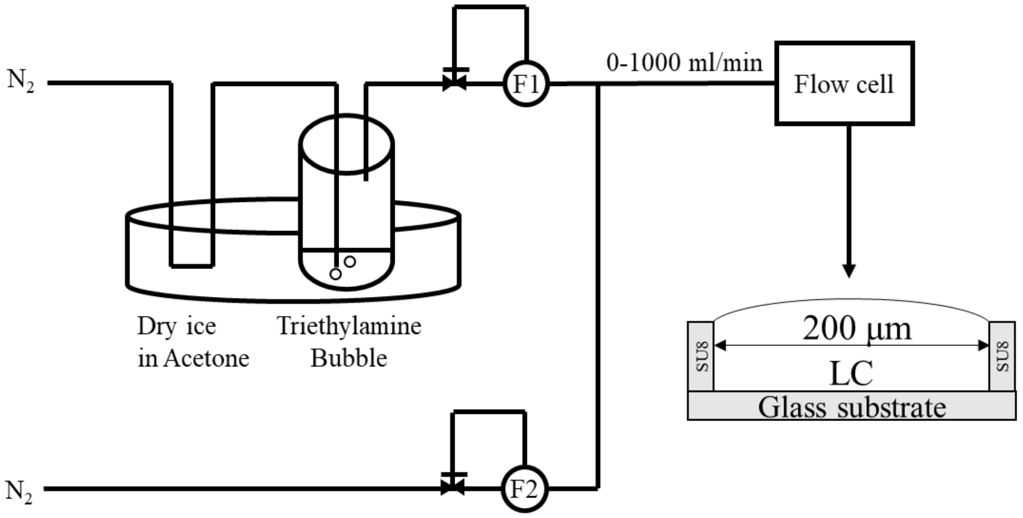

Figure S1. Schematic illustration of the flow cell used to expose a supported LC film to a gas stream at specified flow rate, concentration of TEA.

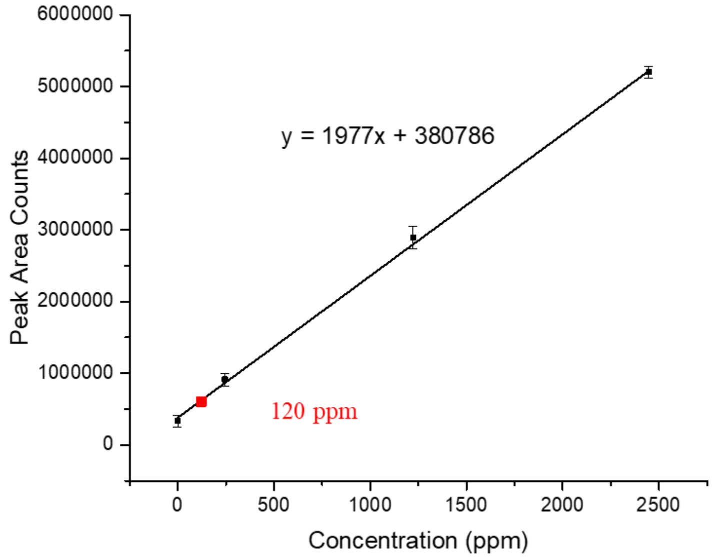

Figure S2. Relationship between TEA concentration and gas chromatography signal. The red dot indicates the concentration flowing in the F1 stream.

Table S1. Transition temperatures (T, °C) and enthalpies (ΔH, J/g) of LCs obtained by DSC in the heating cycle.

LCs	T_{Cry-N}	ΔH_{Cry-N}	T_{N-Iso}	ΔH_{N-Iso}
C4BA	101.6	53.6	114.9	3.7
C5CA	54.8	101.1	105.4	6.8
C4BA+C5CA (50/50 mol%)	30.6	81.4	111.7	4.7
Humid air (80% RH) exposed C4BA+C5CA (50/50 mol%)	30.1	79.3	111.7	3.3
C4BA+C5CA (25/75 mol%)	29.6, 42.1	37.6, 22.9	108.9	4.4

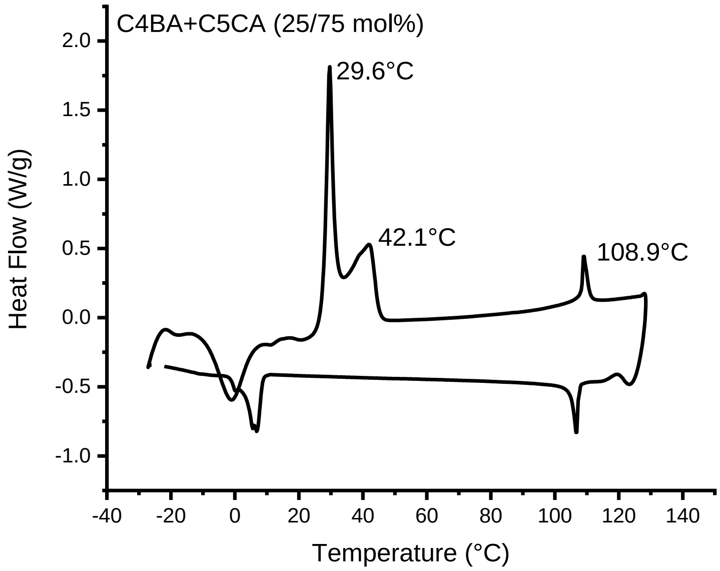

Figure S3. Differential scanning calorimetry (DSC) plots of a mixture of 25 mol% C4BA and 75 mol% C5CA. The upper line corresponds to heating and the bottom line to cooling. DSC scan rate was 5 °C/min.

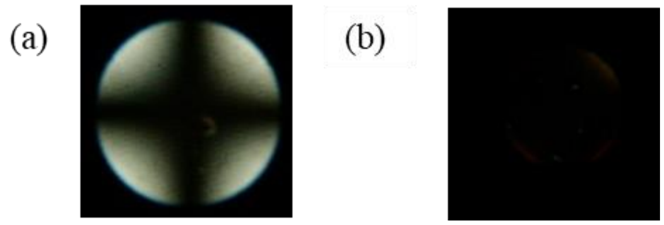

Figure S4. Conoscopic polarized light micrographs (crossed polarizers) of (a) a LC film with a uniform homeotropic orientation, (b) a film of the isotropic 1:1 mixture after TEA (12 ppm) exposure.

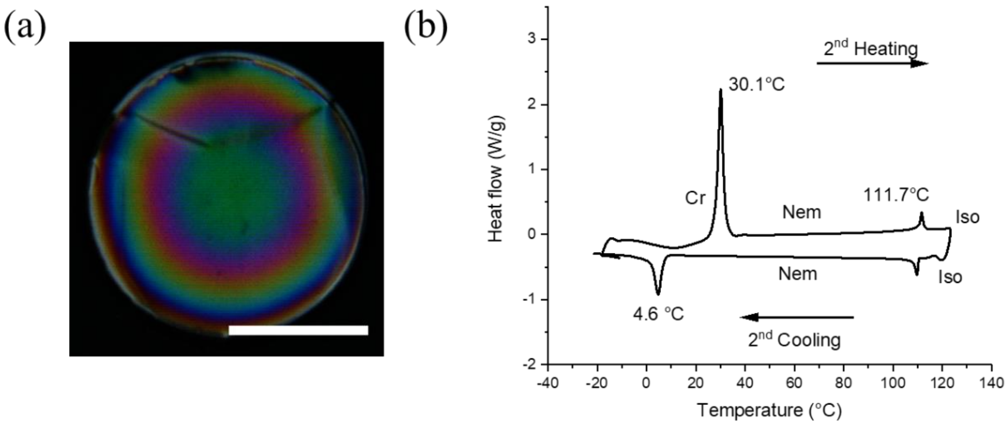

Figure S5. (a) Optical micrographs of representative microwells containing the C4BA+C5CA mixture after exposure to 80% RH air (same as initial state). (b) Differential scanning calorimetry (DSC) plots of 50 mol% C4BA and 50 mol% C5CA mixture after exposure to humid air. The upper line corresponds to heating and the bottom line to cooling. Scale bar: 100 μm. DSC scan rate 5 °C/min.

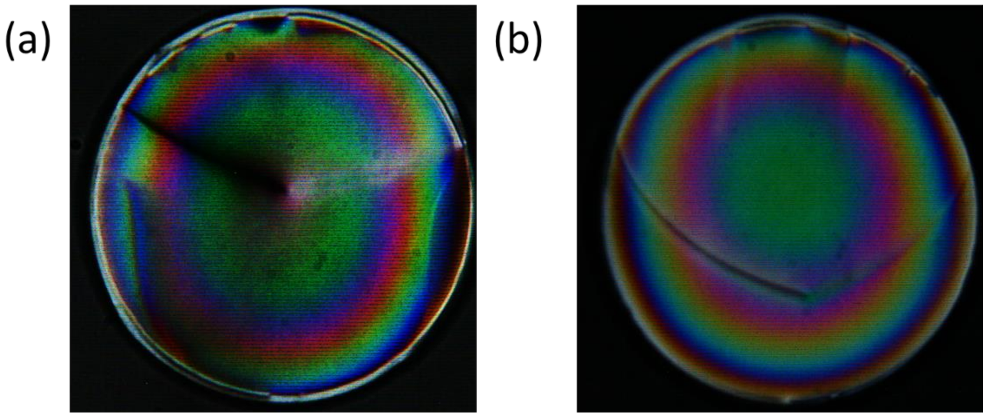

Figure S6. Optical micrographs (crossed polars) of representative microwells containing the C4BA+C5CA mixture (a) after contacting fresh fish (b) after contacting a paper towel saturated with water. The final states of both samples were indistinguishable from their initial states.
